# Long-term care cost drivers and expenditure projection to 2036 in Hong Kong

**DOI:** 10.1186/1472-6963-9-172

**Published:** 2009-09-24

**Authors:** Roger Y Chung, Keith YK Tin, Benjamin J Cowling, King Pan Chan, Wai Man Chan, Su Vui Lo, Gabriel M Leung

**Affiliations:** 1 School of Public Health, Li Ka Shing Faculty of Medicine, The University of Hong Kong, Hong Kong SAR, PR China; 2 Family and Elderly Health Services, Department of Health, Government of the Hong Kong SAR, PR China; 3 Research Office, Food and Health Bureau, Government of the Hong Kong SAR, PR China; 4 Strategy and Planning Division, Hospital Authority, Hong Kong SAR, PR China

## Abstract

**Background:**

Hong Kong's rapidly ageing population, characterised by one of the longest life expectancies and the lowest fertility rate in the world, is likely to drive long-term care (LTC) expenditure higher. This study aims to identify key cost drivers and derive quantitative estimates of Hong Kong's LTC expenditure to 2036.

**Methods:**

We parameterised a macro actuarial simulation with data from official demographic projections, Thematic Household Survey 2004, Hong Kong's Domestic Health Accounts and other routine data from relevant government departments, Hospital Authority and other LTC service providers. Base case results were tested against a wide range of sensitivity assumptions.

**Results:**

Total projected LTC expenditure as a proportion of GDP reflected secular trends in the elderly dependency ratio, showing a shallow dip between 2004 and 2011, but thereafter yielding a monotonic rise to reach 3.0% by 2036. Demographic changes would have a larger impact than changes in unit costs on overall spending. Different sensitivity scenarios resulted in a wide range of spending estimates from 2.2% to 4.9% of GDP. The availability of informal care and the setting of formal care as well as associated unit costs were important drivers of expenditure.

**Conclusion:**

The "demographic window" between the present and 2011 is critical in developing policies to cope with the anticipated burgeoning LTC burden, in concert with the related issues of health care financing and retirement planning.

## Background

**Figure 1:**
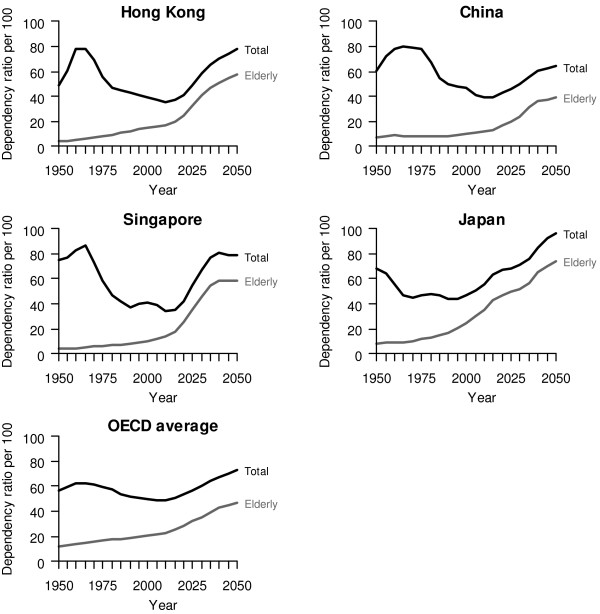
**Projected elderly, total dependency ratios based on official United Nations statistics, 1950-2050 **[39].

Among developed economies, there have been progressively vocal concerns expressed about how to fund long-term care (LTC) for their ageing populations, given generally low fertility rates which are only partly compensated for by immigration [1-5]. This problem is particularly acute in Hong Kong because its fertility rate is the lowest on a sustained basis [6] and its life expectancy is one of the longest in the world [7]. People aged 65 or over will increase by 176% in the next 30 years to 2036, while individuals aged 80 or over will rise even more rapidly by 277% within the same period [8]. This is inevitable for Hong Kong as large birth cohorts of baby boomers plus those born to the large migration waves of young workers during the 1950s and 1960s reach old age over the period [9]. The gravity of the potential burden becomes immediately apparent from inspecting the comparison of total and elderly dependency trends with China, Singapore, Japan and the average OECD countries in Figure 1.

It remains unclear, however, how these changing demographic patterns would translate into expenditure estimates, which in turn are critical for medium to long term budgetary planning and policy responses. In addition, what are the key drivers of LTC spending and would they be amenable to policy interventions? Hong Kong has yet to develop, or begun public discussion of, a comprehensive system-wide response to the potential funding pressures and service provision gap. Nevertheless, in 1997 in the first policy address of the Chief Executive since the repatriation, the government earmarked "care for the elderly" as a major strategic initiative and established the statutory Elderly Commission. Since then, there have been a number of policy initiatives during the past decade, such as the implementation of a standardised care needs assessment, based on the minimum dataset (MDS) instrument, to derive an objective priority list of elders eligible for community-based and institutional services, and the contracting out of residential care services to achieve better efficiency although the quality of such have been the subject of much debate and controversy. Here we offer a first systematic, quantitative examination of the extent of the problem now and in the future.

**Figure 2:**
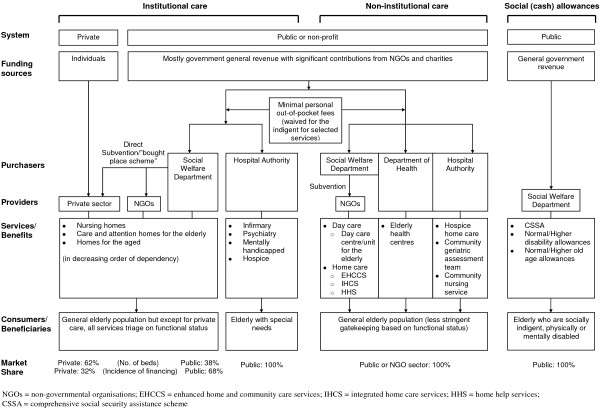
**An overview of Hong Kong's long-term care system**.

Strictly speaking Hong Kong does not have an LTC "system", as opposed to a patchwork of services in having to deal with sporadic needs of the aged population as and when they arose historically; although there have been a myriad of sub-system level internal needs assessment exercises and external consultancy reports over the years. Figure 2 summarises the structural ecology of LTC in Hong Kong currently. Institutional care has traditionally been mostly provided by the public or non-profit sector, the latter directly subvented by the former, although under the more recently introduced "bought place" scheme, private operators in addition to non-governmental organisations (NGOs) have been contracted to provide beds. The publicly financed Hospital Authority (HA), responsible for over 90% of total health care bed-days in the territory, also provides long-stay infirmary, psychiatry, mentally handicapped and hospice beds (see also Table 1). Non-institutional services fall under the remit of the government Social Welfare Department (SWD) and subvented NGOs (providing home care and day care), the Department of Health (DH) (providing primary preventive care at district level elderly health centres), and the HA (providing hospice care and community geriatrics outreach care by both medics and nurses, the latter two also covering older adults residing in institutions). Finally direct (mostly cash) subsidies of various types - socially indigent, physically or mentally disabled, demographically defined (Table 1) - are available to those eligible within and outside institutions.

**Table 1:** Long-term care services, corresponding units of utilisation and covariables in regressions to predict service use

**Services or Allowances**	**Unit of utilisation**	**Covariables (if applicable)**
***Institutional***		
Social Welfare Department/NGO		
• Nursing homes	No. of recipients	Age group, sex, MDS
• Subvented Home for the Aged (H/A)	No. of recipients	Age group, sex, MDS
• Subvented Care and Attention Homes for the Elderly (C&A)	No. of recipients	Age group, sex, MDS
Private sector/NGO		
• Self-financed H/A	No. of recipients	Age group, sex, MDS
• Self-financed C&A	No. of recipients	Age group, sex, MDS
• Private homes	No. of recipients	Age group, sex, MDS
Hospital Authority		
• Long-stay Infirmary	No. of patients	Age group, sex, MDS
• Long-stay Psychiatry	No. of bed days occupied	Not covered by THS 2004
• Long-stay Mentally Handicapped	No. of bed days occupied	Not covered by THS 2004
• Log-stay Hospice	No. of bed days occupied	Not covered by THS 2004
		
***Non-institutional***		
Social Welfare Department/NGO		
• Home Care	No. of recipients	Age group, sex, MDS, marital status,
◦ Enhanced Home and Community Care Services		household composition, housing tenure, housing type, monthly household income, and
◦ Integrated Home Care Services		education
◦ Home Help Services		
• Day Care	No. of recipients	Age group, sex, MDS, marital status,
◦ Day Care Centre/Unit for the Elderly		household composition, housing tenure, housing type, monthly household income, and education
		
Department of Health		
• Elderly Health Centre	No. of attendances	Age group, sex, MDS, marital status, household composition, housing tenure, housing type, monthly household income, and education
		
Hospital Authority		
• Hospice Home Care	No. of visits	Not covered by THS 2004
• Community Medical Services		
◦ Community Geriatric Assessment Team	No. of visits (subvented homes + private H/A)	Not covered by THS 2004
◦ Community Nursing Service	No. of visits	Not covered by THS 2004
		
***Social allowances provided by Social Welfare Department***		
Comprehensive Social Security Assistance (CSSA) Scheme		
• Institutional population	No. of allowance recipients	Age group, sex, MDS
• Non-institutional population	No. of allowance recipients	Age group, sex, MDS, marital status, household composition, housing tenure, housing type, monthly household income, and education
		
Higher Disability Allowance		
• Institutional population	No. of allowance recipients	Age group, sex, MDS
• Non-institutional population	No. of allowance recipients	Age group, sex, MDS, marital status, household composition, housing tenure, housing type, monthly household income, and education
		
Normal Disability Allowance		
• Institutional population	No. of allowance recipients	Age group, sex, MDS
• Non-institutional population	No. of allowance recipients	Age group, sex, MDS, marital status, household composition, housing tenure, housing type, monthly household income, and education
		
Higher Old Age Allowance		
• Institutional population	No. of allowance recipients	Age group, sex, MDS
• Non-institutional population	No. of allowance recipients	Age group, sex, MDS, marital status, household composition, housing tenure, housing type, monthly household income, and education
		
Normal Old Age Allowance		
• Institutional population	No. of allowance recipients	Age group, sex, MDS
• Non-institutional population	No. of allowance recipients	Age group, sex, MDS, marital status, household composition, housing tenure, housing type, monthly household income, and education

Abbreviations

NGO = non-governmental organisations; MDS = validated Chinese version of Minimum Data Set-Home Care; THS = Thematic Household Survey

Categorisation of covariables

Age group (year): 60-64, 65-69, 70-74, 75-79, 80+;

Sex: M, F;

MDS: 1 (No impairment, no health problem), 2 (No impairment with health problem), 3 (Mild impairment, no health problem), 4 (Mild impairment with health problem), 5 (Moderate impairment, no health problem), 6 (Moderate impairment with health problem), 7 (Severe impairment, no health problem),

8 (Severe impairment with health problem);

Marital status: Separated/Divorced/Widowed, Single, Married/Cohabited;

Household composition: Living alone, Not living alone;

Housing type and tenure: Government housing - rented, Government housing - owned, Private housing - rented, Private housing - owned;

Monthly household income (HK$): <5,000, 5,000 - 12,499, 12,500 - 24,999, >24,999;

Education: No schooling/informal schooling, Primary, Secondary/Matriculation, Tertiary/above

In terms of financing source or agent for LTC, unlike say Japan (an East Asian, albeit much larger, neighbour also with a rapidly ageing/aged demographic structure and at a similarly advanced stage of economic development) with a social insurance model of LTC or Singapore (a city-state like Hong Kong with just over half its population size which runs an opt-out "Eldershield" severe disability insurance programme to benefit those at least 65 years, funded through debits from the population-wide medical savings accounts), the incidence of funds to support LTC services comes from government general revenue on the public side and direct out-of-pocket spending for privately purchased care, in approximately a 9:1 ratio [10,11].

Projections of LTC need, demand and associated expenditure have been conducted in various national settings [1,12-17] and by international organisations such as OECD [2], using a range of different methodologies. Two general categories of techniques have prevailed. One approach is to use a state-transition Markov model to simulate the experience of a cohort of older adults as they transition through different health and disability states at regular time intervals, according to a predefined set of transition probabilities [5] parameterised by longitudinal (panel) data [4,18]. However, Hong Kong does not have the requisite individual-level panel information [18]. On a conceptual level, Hong Kong's older population was very recently formed mostly by migrants during and after the Second World War. In fact over 80% of those aged at least 65 years were born in mainland China, whereas most birth cohorts since the 1960s were locally born. Therefore, the inherent historical heterogeneity of different cohorts of older adults presents additional difficulties to adopting the Markovian approach. An alternative methodology relies on the demographic extrapolation of current and projected future needs, while explicitly acknowledging the impact of local epidemiologic transition. One of the most comprehensive and policy-relevant projection models based on this approach is the study by the Personal Social Services Research Unit (PSSRU) in the UK [1]. With the requisite data available in the local context, we adopted this approach to project Hong Kong's total LTC expenditure to the year 2036.

In the following sections, we first detail the actuarial methods underlying the projection model, then summarise empirical results and finally draw conclusions that are directly relevant to policymaking in terms of future health and LTC financing reform.

## Methods

### Definitional boundaries

We considered LTC services according to the OECD definition [2], which refers LTC to a range of often basic services needed for persons who are dependent on help for carrying out basic activities of daily living. LTC aims at making the current unwell condition more bearable. It included health and social care, social security benefits as well as assessment and care management relevant to meeting LTC needs. Table 1 sets out the different types of LTC services and allowance benefits included.

We focused on older adults aged at least 60 years.

**Data **[see Additional File 1: Supplementary Table S1 for details] Baseline utilisation levels of LTC services were variously estimated from the Thematic Household Survey 2004 (THS 2004; N_institutional _= 4114, N_non-institutional _= 4812, where sampling weights were applied in the analysis to represent the entire 60+ population as at 2004) that was specially commissioned to examine LTC issues, and from data provided by the SWD, HA, DH and relevant NGOs. Actual and projected population profiles were obtained from the *Hong Kong Annual Digest of Statistics *[19] and *Hong Kong Population Projections 2007-2036*, both published by the Government Census and Statistics Department. Baseline unit costs and allowances were derived from the latest set of Hong Kong's domestic health accounts [20], government budget estimates, as well as relevant government departments and organisations as above.

For the purposes of international comparability to other countries' expenditure estimates and inter-temporal comparability of the proportion of gross domestic product (GDP) spent on LTC within the local context, we expressed all expenditure estimates as a proportion of GDP. The predicted annual growth of per capita GDP was derived from internal projected estimates of the Financial Services and the Treasury Bureau, as at the first quarter of 2006.

### Start and end dates

We specified the base year to be 2004 given the time of administration of the Thematic Household Survey and the most recent cost estimates derived for that year. Projected estimates were to the year 2036, to coincide with the availability of official demographic projections.

### The projection model

#### Conceptual basis

We took a macro simulation approach following the UK PSSRU's LTC projection model [1,21]. We estimated three key linked outcomes that determine LTC spending: 1) the future numbers of older people requiring LTC; 2) the likely level of demand for LTC services and disability benefits for older people; and 3) the costs associated with this demand (inflated to the year to which the projection year relates). The PSSRU long-term care (LTC) model [1] estimates total LTC expenditure, *E*_*t*_, for each year *t *using the following equation:

(1)

where



*SERNO*_*jt *_represents the projected utilisation volumes in year *t *for service *j (j *= 1 to *k*). *c*_*jt *_is the unit cost of the care service inflated to the year *t*. *p*_*ij *_is the probability of a person in age group-sex profile *i *(*i *= 1 to *g*) receiving service *j*, while *n*_*i *_is the number of older persons in the corresponding profile *i*.

#### Statistical details

For services that were covered by THS 2004, we specified separate multivariable logistic regression equations for each service (Table 1) to estimate the age-sex specific probability of receiving such, as follows:

(2)

where *Y*_*j *_is the utilisation for service *j*, *x*_*c *_(*c *= 1 to *q*) are covariables, and *a*_*c *_the parameter of the corresponding covariables. Covariables considered were based on the PSSRU framework, model fit and parsimony of the regression equations, and also dictated by data availability. They included age group, sex, the validated Chinese version of Minimum Data Set-Home Care (MDS-HC) as a proxy for disability [22], co-habitation pattern (viz marital status and household composition), housing type, housing tenure, and financial means (viz monthly household income and education attainment). Data of these covariables were derived from THS 2004, their descriptive statistics are presented in Additional File 1: Supplementary Table S2, and their distributions per age group and sex are presented in Additional File 1: Supplementary Table S3. The predicted probability of service utilisation associated with each subject, , is derived from the following equation:

(3)

where *x*'s are covariables, and 's their corresponding parameter estimates.

To derive the future numbers of older people requiring LTC, we took the official projected demographic estimates and populated ten age-sex strata: (60-64, male); ...; (80+, male); (60-64, female); ...; (80+, female). We then multiplied the probability of use, as estimated above, by the number of older adults in each stratum, yielding age-sex-service specific utilisation volume as an output for the likely level of demand for LTC services and disability benefits for older people. We assumed constant age-sex-specific intensities of utilisation; thus, the level of demand for LTC services and disability benefits incorporated both the probability and intensity of utilisation. We adjusted for under-/mis-reporting and telescoping [23] by calibrating the predicted volume of service to match observed utilisation patterns (as obtained from relevant government departments) by age group and sex, and applied the resulting correction factors estimated for the base year to all subsequent years. Since our estimation of LTC demand was based on utilisation volumes of the formal LTC service recipients, it did not necessarily include all people who are in need of LTC. To project the unit costs of the services for 2005 to 2036, annual growth in unit costs were set at the average historical rates of change over the period 1999 to 2006. Finally in estimating the associated expenditure, we multiplied the relevant age-sex-specific unit cost by the predicted volume of receiving each service.

We assessed model fit of the regression equations by area under the receiver operating characteristics (ROC) curves and where possible we tried to specify the most parsimonious model. We performed internal validation of each regression model by bootstrapping, where we ran 1,000 iterations by sampling with replacement [Additional File 1: Supplementary Table S4].

For services that were not covered by THS 2004, mostly relating to assessment and care management as well as HA services, we carried out the projection exercise based on past trends of utilisation and unit costs obtained from relevant government departments. Specifically, spending on assessment and care management for the base year was estimated as per the government budgetary subheads of "Services for the Elderly" and "Social Security" programmes of SWD. To project such expenditure for future years, we accorded the same growth rate as that of the number of disabled older people [1], in turn a function of secular demographic change and the associated MDS-HC scores.

For services provided by the HA, we first derived the likely number of HA service recipients, by type, pro rata to the total projected number of older adults, as per the average ratios during 2004 to 2006. Growth in unit costs, by service type, was projected based on the historical rates of change from 1993 to 2004.

Additional File 1: Supplementary Table S5 describes the distribution of utilisation rates of LTC services per age group and sex, whereas Additional File 1: Supplementary Table S6 shows the average unit costs per year of the services.

### **Projection model assumptions and sensitivity scenarios **

[see Additional File 1: Supplementary Table S7 for details]

#### Base case

We assumed that the age-sex-specific probability of service utilisation would remain constant as per the base year of 2004. Real annual growth in unit costs, based on historical trends over the period 1999 to 2006, were respectively set at 2.44%, 4%, 3.5%, and 2% for SWD institutional services, HA services, SWD non-institutional services, and social allowance benefits.

Based on the parameters of the base case, we also tested three hypothetical scenarios by varying the unit cost growth rate and/or population size and structure *ceteris paribus*. They are the "demographic change only," "unit cost change only," and "neither demographic nor unit cost change" scenarios. In the "demographic change only" scenario, we isolated the effect of an ageing and growing population (i.e., demographic changes according to the government population projections by age and sex) by setting the unit cost growth parameters to zero relative to other goods and services in the general economy; i.e., assuming neither utilisation patterns nor relative unit costs change from the baseline in 2004. In the "unit cost change only" scenario, in contrast, we isolated the effect of relative unit cost changes by assuming that the population size and structure would not change from baseline. Finally, in the "neither demographic nor unit cost change" scenario, we estimated total LTC spending assuming no change in the population or relative cost from 2004. Clearly these scenarios are implausible in reality but can illustrate the relative contributions of demography and relative cost structure as spending determinants.

#### Demographic effect

We tested the robustness of the model projections to changes in household composition and marital status.

Given increasingly prevalent trends of older adults living alone that are likely to continue into the future [19,24], we assumed that the proportion of singleton households would reach 12% among males and 18% among females aged 70-74 years by 2036. The rate of change was linearly averaged out over the intervening years. The corresponding proportions for the other age-sex groups were scaled pro rata relative to their association with the 70-74 reference group in 2004.

Empirical observations suggest significant changes in marriage and divorce patterns that may be sustained [24,25]. Accordingly we assumed that among 70-74 men (women), the proportion of being separated/divorced/widowed would drop to 10% (15%), the proportion of being single would reach 10% (10%), thus the proportion of being married would become 80% (75%) by 2036, again implemented evenly over time. Similarly, the corresponding proportions in the other age groups were derived on a pro rata basis relative to the referent 70-74 age categories as at 2004 for each sex separately.

#### Compression of disability

Future secular changes in age-sex specific disability remain controversial [26,27]. On the optimistic side [28], the Brookings assumption specifies that the number of years with disability would remain constant: as life expectancy rises, the number of years without disability would increase by a similar amount. Operationally this involves moving the age-sex-specific disability prevalence forward by one year for each year increase in life expectancy. The double-Brookings scenario assumes that for every additional year of life, disability rates advance by two years; whereas the half-Brookings assumption only shifts the disability rate by half a year.

#### Informal care shift

Informal care refers to assistance given by spouses, other household members, relatives outside the household, neighbours, friends and domestic helpers. We explored the substitutional effect of informal care and the impact of the changing availability of such [29]. Of note, we do not attempt to monetarise the value of informal care or of its opportunity costs [29,30].

We assumed the availability of informal care would decline over time as the labour participation rate increases and traditional family structures and values move away from direct LTC provision within the family context. We incrementally reduced the number of individuals receiving informal care by 0.5, 1, and 2% annually and substituted for this decline by increasing the use of institutional and non-institutional services, matched to level of need as measured by the MDS-HC score according to a matching algorithm set forth by SWD as per current practice [31,32]. There were two matching schemes for the shift to non-institutional services, while there were three matching schemes for the shift to institutional services [see Additional File 1: Supplementary Table S7 for details].

#### Residential to community care shift

To reflect secular trends in deinstitutionalisation, we substituted an equivalent number of individuals who would otherwise be in institutional care with community-dwelling persons requiring formal non-institutional services. We reduced the number receiving institutional care by 1, 2, and 3% every 10 years. The compensatory services affected by such were similarly estimated based on the MDS-HC disability matching scheme [31,32]. There were six matching schemes as detailed in Additional File 1: Supplementary Table S7.

#### Carer-blind

This scenario supposes the same level of formal LTC service receipt regardless of the availability of informal care, which in effect reduces reliance on home or self help. Therefore we assumed that the proportion who would be "living alone" to gradually (i.e., by uniform time-dependent increments) reach 100% by 2036 for all age groups and both sexes.

#### Cost-pressure

Given the highly labour intensive nature of LTC, where technology-driven productivity gains are likely limited, we treated changes in unit costs with the 'Baumol effect' such that relative prices tend to rise compared to other goods and services in the general economy [33]. We increased the annual percentage growth of unit cost by an additional 0.5% (scenario 1) and 1% (scenario 2) over those in the base case.

#### Cost-containment

In contrast, we also explored the effect of constraining the cost pressures as described, likely exerted through public sector supply side measures. Correspondingly we decreased the annual percentage growth by 0.5% (scenario 1) and 1% (scenario 2) below the base case's growth rates.

#### Income elasticity

It is plausible that income growth in the economy could push up LTC expenditure due to greater demand for higher quality services. However, empirical evidence on income elasticity of LTC expenditure is scarce, and is usually assumed to be zero as we did in the base case [2]. Nevertheless, we tested the sensitivity of our results assuming income elasticity of 0.25 (scenario 1), 0.5 (scenario 2), and 1 (scenario 3) based on annual per capita GDP growth.

Additional File 1: Supplementary Table S7 summarises the assumptions underlying the base case, the hypothetical scenarios, and all sensitivity analyses.

All analyses were implemented in R version 2.5.0. All monetary values are expressed in real (i.e., inflation-adjusted) terms in 2004 dollars.

## Results

**Figure 3:**
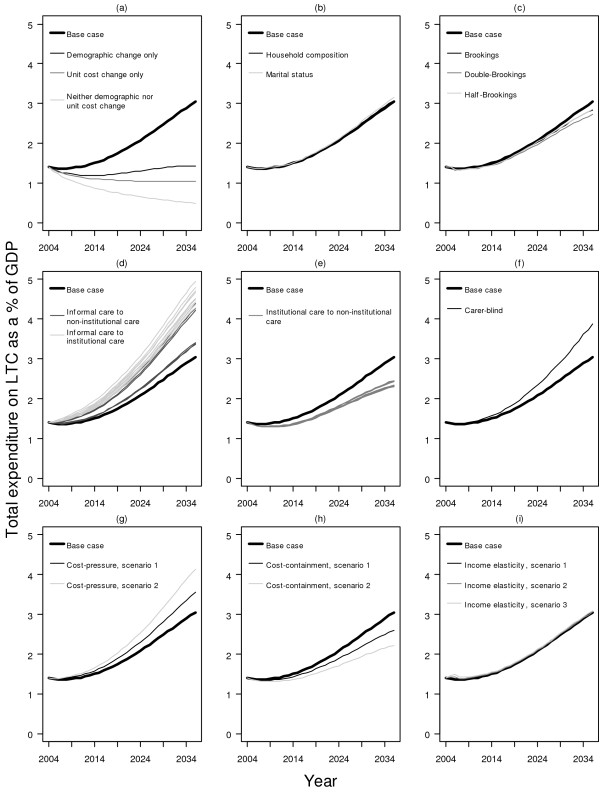
**Estimated total expenditure on long-term care as a proportion (%) of GDP**. Sub-figure: (a) base case + three hypothetical scenarios, (b) base case + demographic effect, (c) base case + compression of disability, (d) base case + informal care shift, (e) base case + institutional care shift to non-institutional care, (f) base case + carer-blind, (g) base case + cost-pressure, (h) base case + cost-containment, (i) base case + income elasticity.

Figure 3 shows the main results of predicted total spending on LTC to the year 2036 for the base case, the hypothetical scenarios and the different sensitivity scenarios. The secular trend for all models reflects changes in the elderly dependency ratio over time (Figure 1), except for hypothetical scenarios 2 and 3, which disregarded demographic changes. The comparative results of the base case and the hypothetical scenarios demonstrate that demographic changes have a larger impact than changes in unit costs on overall LTC expenditure. Specifically, as a proportion of GDP, it was predicted to increase from 1.4% in 2004, with a very temporary reprieve due to the demographic window until 2011, to 3.0% in 2036.

**Figure 4:**
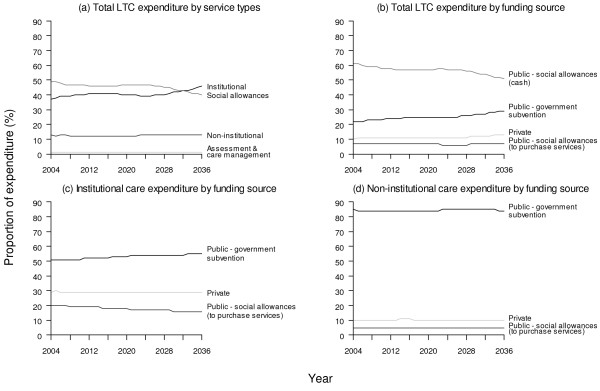
Projected proportions of expenditure for base case every ten years from 2004 to 2034*. Sub-figure: (a) total long-term care by service types, (b) total long-term care by funding source, (c) institutional care by funding source, (d) non-institutional care by funding source. * Projected proportions of expenditure for social allowances and assessment & care management are not shown because they are respectively 100% public and 100% public throughout the projection period.

In relative terms by service mix, the proportion allocated to institutional services is projected to increase from 37% in 2004 to 46% in 2036, while social allowance benefits would correspondingly decline from 49% to 40% during the same interval. Of note, whereas the larger proportion of social allowances are encashed, a smaller portion goes towards paying for institutional and non-institutional care directly. The proportion allocated to non-institutional services is projected to remain steady at around 13%. Figure 4 also shows that funding source (private vs. public) is highly correlated with type of service.

Taken together, the various sensitivity scenarios yielded a range of spending estimates from 2.2% to 4.9% of GDP by 2036 (Figure 3). The highest estimates resulted from assuming the number of older adults receiving informal care would decrease by 2% annually which was substituted by a corresponding increase in government subvented institutional services. In fact, in all instances where the availability of informal care was reduced, LTC spending rose to varying degrees (i.e., progressively more expensive from "carer-blind" to substitution by non-institutional services and institutional care). In contrast, total spending could however become as low as 2.2% (2.6%) of GDP in 2036 if costs were to be controlled to the extent that it would grow less rapidly than productivity gains in the general economy by 1% (0.5%). Similarly, LTC spending could be contained between 2.3% and 2.5% if deinstitutionalisation, viz substitution by home care and day care, would prove successful.

In comparison, changes in unit costs under the 'cost-pressure' scenarios resulted in less variance from the base case. Total LTC expenditure per GDP would reach 3.5% and 4.1% by the end of the projection period respectively for average annual marginal unit cost growth of 0.5% and 1%.

Changes in household composition, marital status or compression of disability, to the extent of our sensitivity analyses [Additional File 1: Supplementary Table S7], made little difference to the projected estimates. Similarly, for scenarios with income elasticities of 0.25, 0.5 and 1, total spendings were all projected to be only slightly above the base case's through to 2036.

## Discussion

Our findings show that, in parallel to secular changes in the elderly dependency ratio, Hong Kong's total spending on LTC would decrease from 1.4% of GDP in 2004 until reaching the same spending level by 2011, but thereafter show monotonic increases reaching 3.0% in 2036 (with an average annual growth rate of 3.1%). Thus the demographic window between the present and 2011 affords Hong Kong a critical period to discuss and debate policy options that would address this burgeoning financial burden. Moreover, demographic changes brought about by ageing are more important in driving the growth of LTC expenditure than non-demographic changes such as unit cost growth. Hong Kong's average annual spending growth rate is 1.1% higher (in absolute terms) than the OECD average over the period from 2005 to 2025, reflecting the intensity of ageing demographics locally compared to other high-income economies [34].

We outline some potential caveats. First, ours is an actuarial illustration of plausible scenarios and should not be taken as precise quantitative predictions of the future. Inherent in our linear extrapolations is the disregard of dynamic behavioural changes in response to policy interventions or macro-economic forces otherwise. Numerous other assumptions of the projection model, although explicitly detailed here, limited the range of the results and are all liable to deviations from actual circumstances in the future. Second, a major part of our data was based on THS 2004; thus the usual biases of a population-based survey would apply to our estimates. In mitigation, the predicted utilisation patterns were adjusted with reference to the age group-sex distribution of the observed utilisation patterns to control for under-/mis-reporting. Third, we should ideally have access to longitudinal panel data following a prospective cohort of ageing adults, which would have allowed us to implement an econometric model instead thus being able to take into account the dynamical nature of change. However, even were such available, social changes between a historical cohort and future generations of older adults would likely limit the interpretation of the empirical observations. Fourth, by grouping together all older persons aged 80 or above, the model is unable to disaggregate potential heterogeneity among the oldest-old whose dependency on formal or institutionalised LTC would be particularly acute. However, this was necessitated for the sake of statistical robustness of the model given the very small collective sample size of the 80+ group, lest the stratified cells contain too many zero counts. Fifth, expressing the future LTC financing burden as a proportion of GDP necessarily requires the projection of general economic growth (albeit based on rigorous statistical treatment of historical trends and implemented independently by the Government Economist). This has added an extra layer of uncertainty to the estimates that should be borne in mind when interpreting the results, but which reinforces our first caveat. Last, when considering the dual policy areas of health and LTC financing, there is some double-counting of expenditure due to overlapping definitional boundaries by convention in the two sets of literature. To be specific, these overlaps include all HA services, DH's elderly health centres, nursing homes, day care services, enhanced home and community care, as well as elderly with MDS level 7 or above residing in subvented and self-financed care and attention homes and private homes. They accounted for 31% of the total LTC expenditure in 2004, and our projections indicate that this proportion would rise to 37% by 2036. Acknowledging and accounting for such is especially important as Hong Kong is at present undergoing a consultation exercise on supplementary health financing with particular emphasis on post-65 health care needs [35]. Therefore clear distinction between LTC and health care definitional boundaries must be maintained between two closely related policy areas.

LTC planning should not be treated in isolation from the closely related policy issues of health care financing, where spending tends to be concentrated among the elderly, and retirement planning in general. A thorough and comprehensive examination of all three areas deserves special emphasis in Hong Kong, which has traditionally espoused a self-reliant society, rather than adopting the welfare state model of say continental Europe. While such a laissez-faire approach has allowed it to prosper and lift its largely immigrant population of young workers out of poverty since the 1950s, it poses an enormous challenge to policymakers who now have to deal with the same rapidly ageing individuals as they require health and long-term care and demand a share in the fruits of development.

Currently about one-tenth of LTC spending traces its incidence from private pockets while public sources provide the remaining majority. Looking into the future, given current patterns, public finances appear poised to be further saddled by the projected higher expenditure, not only in absolute but also relative terms. Compared to OECD economies, all of which levy substantially higher and broader-based taxes than Hong Kong, the local public revenue base as currently constituted is unlikely able to sustain this growing financial burden [2]. Coupled with the twinned set of health care expenses, which we projected to grow from 5.2% GDP in 2004 to 9.2% by 2033 [36], recurrent allocations would have to increase from 19.6% to 36.8% of total government budget by 2033 *ceteris paribus *which would crowd out other policy areas requiring public finances such as education, social welfare (except LTC) and security.

The issue of (non-disability related) social allowances, particularly given their large share of total LTC spending, should be considered together with the sufficiency of general retirement savings. Since 2000, Hong Kong has mandated that all workers and employers contribute, up to a cap, 5% of wages to a personal provident fund account from which withdrawal is only allowed post-65. Whether this progressively maturing retirement savings scheme could obviate at least some of the old age allowances currently in place remains to be examined although there would be tremendous political pressure against government withdrawing from such age-based welfare support.

There are several generalisable lessons that may be useful for and from other countries, especially those with a similar demographic and socioeconomic profile (e.g., Switzerland, Israel) and geo-cultural background (e.g., Singapore, Japan and mainland Chinese coastal urban centres). First, whereas there is an increasing tendency for Hong Kong residents to retire across the border in the Pearl River Delta (PRD) region of mainland China [37], the issue of whether such should be encouraged through economic incentives, given the much lower cost base of providing LTC north of the border thereby allowing further containment of total spending, needs further fleshing out and debate. In part this would depend on the long-term geopolitical integration of Hong Kong into the PRD and whether portability of welfare benefits such as post-retirement social and health care are generally extended to the PRD. Second, the 1999 Harvard Report [38] recommended an individual savings account (MEDISAGE), modelled after the Singaporean Medisave and ElderShield programme, to finance LTC in Hong Kong. Although only scant details were provided and no consensus was reached, renewed dialogue on the viability of such a programme should be vigorously pursued before the demographic window closes, and especially in the light of the recent health system reform consultation exercise [35]. Third, different countries have experimented with different ways of raising resources to support LTC. Japan, for example, with one of the longest living populations in the world, has recently mandated LTC social insurance in keeping with the same approach to health financing. This may be an alternative path for Hong Kong to consider although the appealing inter-temporal risk pooling with self inherent in the Singaporean savings account design would be lost.

Strengths of our study include:- 1) implementation of the model adapted from the validated methods of the UK PSSRU team [1,21] and further modified according to OECD specifications for international comparability [2]; 2) parameterisation of the model with locally relevant and up-to-date empirical data, including a recent special survey of representative samples of institutional and community-dwelling older adults; 3) objective needs assessment of and prediction of service use by LTC-related demographic and non-demographic factors such as the validated Chinese version MDS-HC, marital status, household composition, housing tenure and type, monthly household income and educational attainment; 4) robust uncertainty analysis by bootstrapping; and 5) identification of the impact of various "control knobs" or potential cost drivers by testing the base case against a wide range of sensitivity scenarios.

## Conclusion

Two key take-home messages can be drawn from this projection exercise. First, in contrast to health care costs, demographic changes brought about by ageing are more important in driving LTC spending growth than non-demographic changes such as unit cost growth. In short, rapidly-ageing Hong Kong will inevitably bear an increasing LTC burden, unless there is a dramatic change in its population policy vis-à-vis substantially increased immigration of mainland Chinese or mass emigration of retirees. Second, the period between the present and 2011 is critical in developing policies to cope with the LTC burden alongside with the issues of health care financing and retirement planning. Irrespective of the eventual policy responses to Hong Kong's ageing population thus LTC need or demand, the reliable prediction of the associated financial liability in the medium to long run, as presented in this study, adds to the evidence base from which such can begin to be formulated and debated.

## Competing interests

The authors declare that they have no competing interests.

## Authors' contributions

RYC participated in the design of the study, acquisition and analysis of the data, interpretation of the results, drafting and revision of the manuscript. KYKT contributed to the design of the study, acquisition and analysis of the data, interpretation of the results and revision of the manuscript. BJC participated in the design of the study, analysis of the data, and revision of the manuscript. KPC participated in the analysis of the data and revision of the manuscript. WMC and SVL participated in the conception and design of the study, acquisition of the data, as well as revision of the manuscript. GML contributed to the conception and design of the study, acquisition of the data, interpretation of the results, and revision of the manuscript. All authors read and approved the final manuscript.

## Pre-publication history

The pre-publication history for this paper can be accessed here:



## Supplementary Material

Additional file 1 **Supplementary Tables**. This additional file includes 7 tables supplementary to the main text of the manuscript (Supplementary Table S1-S7). Click here for file
