# Supplementary material for: Long-term care cost drivers and expenditure projection to 2036 in Hong Kong
Source: BMC Health Serv Res. 2009 Sep 24;9:172. doi: 10.1186/1472-6963-9-172 (PMC2765433; doi:10.1186/1472-6963-9-172)
Supplement: Additional file 1 — Supplementary Tables. This additional file includes 7 tables supplementary to the main text of the manuscript (Supplementary Table S1-S7). [file 1472-6963-9-172-S1.doc]

**Additional files**

**Additional file 1**

**Supplementary Table S**1. Data sources

| **Source** | **Year(s) of Publication** | **Data** |
| --- | --- | --- |
| Thematic Household Survey (THS) (Census and Statistics Department) | 2004 | Current prevalence and distribution of older adults living in domestic household (by household type), or living in institutions, using informal care, formal non-institutional care, or formal institutional care; comprehensive needs assessment with a modified MDS-HC instrument and associated scoring algorithm validated in the local context |
| *Hong Kong Population Projections, 2007-2036* (Census and Statistics Department) | 2007 | Projected numbers of older adults by age and sex |
| *Hong Kong Annual Digest of Statistics* (Census and Statistics Department) | 2004, 2005, 2006 | Actual numbers of older adults by age and sex |
| Hong Kong’s Domestic Health Accounts (DHA) | 1989/90 – 2004/05 | Utilisation as well as aggregate and unit costs of different types of LTC services that fall under the definitional boundaries of health care under the OECD *A System of Health Accounts* framework |
| Hong Kong Government Budget Estimates (Treasury) | 2003/2004, 2004/2005 | Utilisation and unit costs of different types of LTC services provided by NGOs with subvention by SWD, HA, and DH |
| Social Welfare Department, Hospital Authority, and NGOs | Current + historical trends | Utilisation as well as aggregate and unit costs of different types of LTC services (particularly those unavailable from the DHA), including assessment and care management and social security allowances |

SWD=Social Welfare Department

HA=Hospital Authority

DH=Department of Health

NGO=non-governmental organisations

**Supplementary Table S2. Summary statistics of the covariables used in the logistic regression models**

| **Variables** | **Categories** | **Counts (%)** |
| --- | --- | --- |
| Age group | 60-64  65-69  70-74  75-79  80+^§^ | 1,256 (14.07%)  1,547 (17.33%)  1,548 (17.34%)  1,326 (14.86%)  3,249 (36.40%) |
| Sex | M^§^  F | 4,153 (46.53%)  4,773 (53.47%) |
| MDS | 1^§^  2  3  4  5  6  7  8 | 3,704 (14.50%)  196 (2.20%)  943 (10.56%)  102 (1.14%)  1,023 (11.46%)  194 (2.17%)  2,386 (26.73%)  378 (4.23%) |
| Marital status | Separated/Divorced/Widowed^§^  Single  Married/Cohabited | 1,466 (30.47%)  120 (2.49%)  3,226 (67.04%) |
| Household | Not living alone^§^  Living alone | 4,287 (89.09%)  525 (10.91%) |
| Housing type and tenure | Government housing – rented^§^  Government housing – owned  Private housing – rented  Private housing – owned | 2,155 (44.78%)  729 (15.15%)  285 (5.92%)  1,643 (34.14%) |
| Monthly household income (HK$) | <5,000^§^  5,000 - 12,499  12,500 - 24,999  >24,999 | 1,130 (23.48%)  1,368 (28.44%)  1,215 (25.25%)  1,099 (22.83%) |
| Education | No schooling/informal schooling^§^  Primary  Secondary/Matriculation  Tertiary/above | 4,557 (51.05%)  2,686 (30.09%)  1,307 (14.64%)  376 (4.21%) |

^§^ **= Reference group**

**Supplementary Table S3. Weighted distribution (percentage) of covariables per age group and sex** in Thematic Household Survey 2004

|  |  | **60-64** | | **65-69** | | **70-74** | | **75-79** | | **80+** | |
| --- | --- | --- | --- | --- | --- | --- | --- | --- | --- | --- | --- |
| **Variables** | **Categories** | **M** | **F** | **M** | **F** | **M** | **F** | **M** | **F** | **M** | **F** |
| MDS | 1 | 771  (75.29%) | 721  (77.07%) | 762  (75.15%) | 709  (68.44%) | 627  (68.28%) | 590  (60.31%) | 362  (61.84%) | 437  (56.76%) | 281 (45.84%) | 353  (33.47%) |
|  | 2 | 26 (2.52%) | 6  (0.66%) | 13  (1.30%) | 31  (2.99%) | 29  (3.15%) | 23  (2.37%) | 15 (2.57%) | 20  (2.57%) | 19  (3.12%) | 25  (2.37%) |
|  | 3 | 81 (7.96%) | 100  (10.67%) | 105 (10.40%) | 146  (14.10%) | 125 (13.57%) | 140  (14.36%) | 64 (10.87%) | 116  (15.12%) | 98 (15.94%) | 174  (16.52%) |
|  | 4 | 11 (1.13%) | 7  (0.73%) | 15 (1.49%) | 9  (0.90%) | 3  (0.33%) | 17  (1.74%) | 13  (2.21%) | 20  (2.57%) | 14  (2.36%) | 20  (1.87%) |
|  | 5 | 107  (10.50%) | 72  (7.71%) | 74  (7.31%) | 99  (9.55%) | 82  (8.90%) | 136  (13.90%) | 74  (12.59%) | 97  (12.64%) | 93  (15.23%) | 165  (15.69%) |
|  | 6 | 13  (1.31%) | 17  (1.77%) | 20  (2.02%) | 19  (1.80%) | 14  (1.47%) | 25  (2.52%) | 19  (3.21%) | 24  (3.14%) | 22  (3.59%) | 34  (3.27%) |
|  | 7 | 10  (0.94%) | 8  (0.82%) | 16  (1.61%) | 18  (1.77%) | 32  (3.52%) | 32  (3.24%) | 33  (5.69%) | 40  (5.25%) | 74  (12.07%) | 210  (19.97%) |
|  | 8 | 4  (0.35%) | 5  (0.37%) | 7  (0.72%) | 4  (0.43%) | 7  (0.79%) | 15  (1.55%) | 6  (1.01%) | 15  (1.96%) | 11  (1.86%) | 72  (6.84%) |
| Marital status | Separated/Divorced/  Widowed | 33  (5.55%) | 123  (23.72%) | 68  (11.64%) | 186  (32.60%) | 67  (12.83%) | 235  (44.16%) | 60  (18.61%) | 235  (58.58%) | 86  (27.32%) | 336  (75.21%) |
|  | Single | 19  (3.11%) | 12  (2.22%) | 21  (3.55%) | 8  (1.48%) | 18  (3.45%) | 4  (0.79%) | 6  (2.00%) | 6  (1.60%) | 9  (2.73%) | 16  (3.60%) |
|  | Married/Cohabited | 543  (91.34%) | 385  (74.06%) | 496  (84.82%) | 377  (65.92%) | 436  (83.72%) | 293  (55.05%) | 257  (79.39%) | 160  (39.82%) | 221  (69.96%) | 95  (21.19%) |
| Household | Not living alone | 555  (93.31%) | 492  (94.72%) | 532  (90.92%) | 522  (91.25%) | 467  (89.69%) | 473  (88.89%) | 287  (88.53%) | 336  (83.68%) | 281  (89.21%) | 352  (78.75%) |
|  | Living alone | 40  (6.69%) | 27  (5.28%) | 53  (9.08%) | 50  (8.75%) | 54  (10.31%) | 59  (11.11%) | 37  (11.47%) | 66  (16.32%) | 34  (10.79%) | 95  (21.25%) |
| Housing type and tenure | Government housing – rented | 230  (38.73%) | 190  (36.66%) | 234  (40.00%) | 223  (39.01%) | 246  (47.20%) | 204  (38.43%) | 140  (43.24%) | 157  (39.13%) | 118  (37.39%) | 159  (35.61%) |
|  | Government housing – owned | 79  (13.31%) | 66  (12.71%) | 92  (15.79%) | 92  (16.17%) | 62  (11.94%) | 87  (16.42%) | 43  (13.20%) | 51  (12.63%) | 37  (11.74%) | 54  (12.03%) |
|  | Private housing – rented | 67  (11.22%) | 34  (6.46%) | 40  (6.85%) | 34  (5.97%) | 28  (5.44%) | 27  (5.00%) | 22  (6.88%) | 29  (7.24%) | 16  (4.99%) | 36  (8.02%) |
|  | Private housing – owned | 218  (36.73%) | 229  (44.18%) | 219  (37.36%) | 222  (38.85%) | 185  (35.43%) | 214  (40.15%) | 119  (36.68%) | 165  (41.00%) | 145  (45.88%) | 198  (44.34%) |
| Monthly household income (HK$) | <5,000 | 79  (13.28%) | 82  (15.78%) | 112  (19.14%) | 116  (20.21%) | 130  (24.94%) | 140  (26.30%) | 106  (32.87%) | 125  (31.20%) | 88  (27.76%) | 135  (30.14%) |
|  | 5,000 - 12,499 | 173  (29.03%) | 148  (28.49%) | 159  (27.22%) | 158  (27.53%) | 165  (31.57%) | 135  (25.34%) | 77  (23.91%) | 99  (24.59%) | 94  (29.78%) | 129  (28.76%) |
|  | 12,500 - 24,999 | 158  (26.50%) | 132  (25.39%) | 164  (28.06%) | 140  (24.53%) | 118  (22.69%) | 137  (25.78%) | 81  (25.12%) | 94  (23.35%) | 75  (23.80%) | 104  (23.35%) |
|  | >24,999 | 185  (31.18%) | 158  (30.35%) | 150  (25.58%) | 159  (27.73%) | 108  (20.79%) | 120  (22.58%) | 59  (18.10%) | 84  (20.86%) | 59  (18.65%) | 79  (17.76%) |
| Education | No schooling/informal schooling | 93  (9.12%) | 230  (24.56%) | 135  (13.30%) | 404  (38.96%) | 248  (26.99%) | 531  (54.26%) | 211  (36.10%) | 503  (65.26%) | 267  (43.55%) | 749  (71.07%) |
|  | Primary | 388  (37.95%) | 333  (35.59%) | 444  (43.82%) | 401  (38.76%) | 423  (46.09%) | 339  (34.62%) | 259  (44.22%) | 191  (24.82%) | 198  (32.32%) | 211  (20.05%) |
|  | Secondary/  Matriculation | 415  (40.58%) | 294  (31.46%) | 314  (31.02%) | 171  (16.52%) | 191  (20.80%) | 84  (8.62%) | 95  (16.17%) | 60  (7.74%) | 98  (15.93%) | 80  (7.62%) |
|  | Tertiary/above | 126  (12.25%) | 78  (8.38%) | 120  (11.87%) | 60  (5.77%) | 56  (6.11%) | 24  (2.50%) | 21  (3.51%) | 17  (2.19%) | 50  (8.19%) | 13  (1.27%) |

**Supplementary Table S4**. Areas under the receiver operating characteristics (ROC) curve and their bootstrapped median, upper and lower bounds for the regression equations of each long-term care service

| **Services or allowances** | **Area under ROC curve** | **Bootstrapped median area under ROC curve** | **Bootstrapped lower bound of area under ROC curve** | **Bootstrapped upper bound of area under ROC curve** |
| --- | --- | --- | --- | --- |
| ***Institutional*** | | | | |
| Social Welfare Department/NGO | | | | |
| - Nursing homes | 0.866 | 0.865 | 0.859 | 0.868 |
| - Subvented Home for the Aged (H/A) | 0.769 | 0.767 | 0.761 | 0.771 |
| - Subvented Care and Attention Homes for the Elderly (C&A) | 0.748 | 0.746 | 0.744 | 0.748 |
| Private sector/NGO | | | | |
| - Self-financed H/A | 0.735 | 0.730 | 0.721 | 0.735 |
| - Self-financed C&A | 0.750 | 0.745 | 0.736 | 0.750 |
| - Private homes | 0.818 | 0.817 | 0.816 | 0.818 |
| Hospital Authority |  |  |  |  |
| - Long-stay Infirmary | 0.868 | 0.865 | 0.856 | 0.870 |
| ***Non-institutional*** | | | | |
| Social Welfare Department/NGO | | | | |
| - Home care | 0.865 | 0.824 | 0.766 | 0.854 |
| - Day care | 0.872 | 0.800 | 0.672 | 0.859 |
| Department of Health | | | | |
| - Elderly Health Centre | 0.617 | 0.603 | 0.591 | 0.612 |
| ***Social allowances provided by Social Welfare Department*** | | | | |
| Comprehensive Social Security Assistance (CSSA) Scheme | | | | |
| - Institutional population | 0.539 | 0.532 | 0.518 | 0.540 |
| - Non-institutional population | 0.904 | 0.901 | 0.898 | 0.902 |
| Higher Disability Allowance | | | | |
| - Institutional population | 0.605 | 0.595 | 0.575 | 0.604 |
| - Non-institutional population | 0.893 | 0.851 | 0.770 | 0.895 |
| Normal Disability Allowance | | | | |
| - Institutional population | 0.629 | 0.618 | 0.593 | 0.628 |
| - Non-institutional population | 0.770 | 0.740 | 0.700 | 0.766 |
| Higher Old Age Allowance | | | | |
| - Institutional population | 0.591 | 0.583 | 0.565 | 0.593 |
| - Non-institutional population | 0.728 | 0.723 | 0.717 | 0.727 |
| Normal Old Age Allowance | | | | |
| - Institutional population | 0.603 | 0.584 | 0.498 | 0.603 |
| - Non-institutional population | 0.665 | 0.655 | 0.646 | 0.660 |

**Supplementary Table S5. Weighted distribution (percentage) of LTC services utilis**ation per age group and sex in Thematic Household Survey 2004

|  | **60-64** | | **65-69** | | **70-74** | | **75-79** | | **80+** | |
| --- | --- | --- | --- | --- | --- | --- | --- | --- | --- | --- |
| **Services or allowances** | **M** | **F** | **M** | **F** | **M** | **F** | **M** | **F** | **M** | **F** |
| ***Institutional*** |  |  |  |  |  |  |  |  |  |  |
| Social Welfare Department/NGO |  |  |  |  |  |  |  |  |  |  |
| - Nursing homes | 1  (0.88%) | 0  (0.00%) | 6  (3.82%) | 2  (3.40%) | 12  (3.62%) | 6  (4.03%) | 13  (3.34%) | 13  (3.77%) | 30  (3.45%) | 51  (2.95%) |
| - Subvented Home for the Aged (H/A) | 3  (3.80%) | 1  (7.94%) | 9  (5.86%) | 6  (9.91%) | 29  (9.14%) | 27  (17.34%) | 33  (8.87%) | 50  (14.83%) | 85  (9.69%) | 239  (13.82%) |
| - Subvented Care and Attention Homes for the Elderly (C&A) | 4  (5.22%) | 1  (3.40%) | 24  (15.20%) | 9  (15.63%) | 53  (16.67%) | 25  (16.10%) | 62  (16.72%) | 62  (18.42%) | 185  (21.01%) | 419  (24.22%) |
| Private sector/NGO |  |  |  |  |  |  |  |  |  |  |
| - Self-financed H/A | 0  (0.00%) | 0  (0.00%) | 1  (0.57%) | 1  (1.08%) | 1  (0.45%) | 2  (1.28%) | 3  (0.71%) | 4  (1.29%) | 7  (0.79%) | 31  (1.83%) |
| - Self-financed C&A | 1  (1.65%) | 1  (4.04%) | 2  (1.42%) | 2  (3.11%) | 5  (1.57%) | 4  (2.76%) | 8  (2.19%) | 9  (2.73%) | 23  (2.63%) | 48  (2.79%) |
| - Private homes | 70  (85.02%) | 14  (79.98%) | 112  (70.20%) | 37  (62.66%) | 210  (66.03%) | 89  (56.59%) | 249  (66.87%) | 193  (57.20%) | 539  (61.22%) | 916  (52.94%) |
| Hospital Authority |  |  |  |  |  |  |  |  |  |  |
| - Long-stay Infirmary | 3  (3.18%) | 1  (3.92%) | 5  (2.92%) | 2  (4.22%) | 8  (2.51%) | 3  (1.89%) | 5  (1.29%) | 6  (1.76%) | 11  (1.20%) | 25  (1.46%) |
| ***Non-institutional*** |  |  |  |  |  |  |  |  |  |  |
| Social Welfare Department/NGO |  |  |  |  |  |  |  |  |  |  |
| - Home care | 0  (0.00%) | 0  (0.00%) | 5  (0.83%) | 3  (0.57%) | 1  (0.16%) | 7  (1.25%) | 3  (0.78%) | 4  (0.97%) | 9  (2.97%) | 9  (2.00%) |
| - Day care | 0  (0.00%) | 0  (0.00%) | 2  (0.37%) | 2  (0.40%) | 1  (0.16%) | 3  (0.51%) | 0  (0.00%) | 2  (0.49%) | 5  (1.65%) | 6  (1.26%) |
| Department of Health |  |  |  |  |  |  |  |  |  |  |
| - Elderly Health Centre | 30  (4.98%) | 37  (7.16%) | 59  (10.06%) | 93  (16.30%) | 64  (12.33%) | 83  (15.53%) | 40  (12.25%) | 61  (15.22%) | 32  (10.25%) | 69  (15.51%) |
| ***Social allowances provided by Social Welfare Department*** |  |  |  |  |  |  |  |  |  |  |
| Comprehensive Social Security Assistance (CSSA) Scheme |  |  |  |  |  |  |  |  |  |  |
| - Institutional population | 68  (82.36%) | 14  (79.74%) | 129  (80.81%) | 42  (72.32%) | 252  (79.26%) | 110  (69.84%) | 270  (72.46%) | 247  (73.14%) | 655  (74.43%) | 1335  (77.12%) |
| - Non-institutional population | 52  (8.76%) | 11  (2.17%) | 48  (8.23%) | 29  (4.99%) | 73  (14.07%) | 40  (7.53%) | 36  (11.12%) | 37  (9.16%) | 37  (11.65%) | 47  (10.57%) |
| Higher Disability Allowance |  |  |  |  |  |  |  |  |  |  |
| - Institutional population | 5  (6.64%) | 1  (2.96%) | 8  (5.22%) | 5  (8.47%) | 22  (7.00%) | 12  (7.44%) | 27  (7.25%) | 21  (6.20%) | 54  (6.19%) | 67  (3.87%) |
| - Non-institutional population | 3  (0.47%) | 0  (0.00%) | 1  (0.16%) | 5  (0.80%) | 4  (0.71%) | 1  (0.17%) | 2  (0.52%) | 3  (0.69%) | 6  (1.87%) | 9  (1.98%) |
| Normal Disability Allowance |  |  |  |  |  |  |  |  |  |  |
| - Institutional population | 1  (0.57%) | 1  (6.71%) | 5  (2.96%) | 2  (3.24%) | 13  (3.97%) | 4  (2.67%) | 12  (3.30%) | 10  (2.94%) | 27  (3.11%) | 67  (3.86%) |
| - Non-institutional population | 8  (1.35%) | 4  (0.72%) | 8  (1.31%) | 3  (0.57%) | 11  (2.14%) | 13  (2.44%) | 4  (1.39%) | 5  (1.18%) | 3  (0.96%) | 17  (3.73%) |
| Higher Old Age Allowance |  |  |  |  |  |  |  |  |  |  |
| - Institutional population | 1  (0.35%) | 1  (1.01%) | 3  (2.18%) | 2  (2.73%) | 20  (6.31%) | 21  (13.44%) | 52  (13.91%) | 52  (15.30%) | 111  (12.64%) | 214  (12.34%) |
| - Non-institutional population | 0  (0.00%) | 0  (0.00%) | 22  (3.70%) | 35  (6.19%) | 322  (61.85%) | 360  (67.63%) | 256  (79.14%) | 320  (79.58%) | 245  (77.73%) | 344  (76.86%) |
| Normal Old Age Allowance |  |  |  |  |  |  |  |  |  |  |
| - Institutional population | 1  (1.01%) | 0  (0.00%) | 6  (3.88%) | 5  (8.83%) | 0  (0.00%) | 3  (1.75%) | 0  (0.00%) | 0  (0.00%) | 0  (0.00%) | 0  (0.00%) |
| - Non-institutional population | 0  (0.00%) | 0  (0.00%) | 180  (30.76%) | 221  (38.66%) | 29  (5.59%) | 43  (8.11%) | 4  (1.30%) | 3  (0.83%) | 4  (1.15%) | 5  (1.05%) |

**Supplementary Table S6. Average unit costs (HK$)** per year of LTC services

| **Services or Allowances** | **Unit costs per year** |  |
| --- | --- | --- |
| ***Institutional*** | | |
| Social Welfare Department/NGO | | |
| - Nursing homes | 16,343 / recipient |  |
| - Subvented Home for the Aged (H/A) | 52,474.7 / recipient |  |
| - Subvented Care and Attention Homes for the Elderly (C&A) | 96,035.2 / recipient |  |
| Private sector/NGO | | |
| - Self-financed H/A | 42,228.9 / recipient |  |
| - Self-financed C&A | 102,300 / recipient |  |
| - Private homes | 93,248.1 / recipient |  |
| Hospital Authority | | |
| - Long-stay Infirmary | 145,225 / patient |  |
| - Long-stay Psychiatry | 1,425 / bed day occupied |  |
| - Long-stay Mentally Handicapped | 1,015 / bed day occupied |  |
| - Log-stay Hospice | 2,197.5 / bed day occupied |  |
| ***Non-institutional*** | | |
| Social Welfare Department/NGO | | |
| - Home Care | 20,729.5 / recipient |  |
| - - Enhanced Home and Community Care Services |
| - - Integrated Home Care Services |
| - - Home Help Services |
| - Day Care | 83,280 / recipient |  |
| - - Day Care Centre/Unit for the Elderly |
| Department of Health | | |
| - Elderly Health Centre | 647 / attendance |  |
| Hospital Authority | | |
| - Hospice Home Care | 902.5 / visit |  |
| - Community Medical Services | | |
| - - Community Geriatric Assessment Team | 367.5 / visit |  |
| - - Community Nursing Service | 322.5 / visit |  |
| ***Social allowances provided by Social Welfare Department*** | | |
| Comprehensive Social Security Assistance (CSSA) Scheme | | |
| - Institutional population | 42,036 / recipient |  |
| - Non-institutional population | 42,036 / recipient |  |
| Higher Disability Allowance | | |
| - Institutional population | 25,792.1 / recipient |  |
| - Non-institutional population | 25,792.1 / recipient |  |
| Normal Disability Allowance | | |
| - Institutional population | 12,810.4 / recipient |  |
| - Non-institutional population | 12,810.4 / recipient |  |
| Higher Old Age Allowance | | |
| - Institutional population | 8,097.3 / recipient |  |
| - Non-institutional population | 8,097.3 / recipient |  |
| Normal Old Age Allowance | | |
| - Institutional population | 7,526.0 / recipient |  |
| - Non-institutional population | 7,526.0 / recipient |  |

**Supplementary Table S7. Assumptions for base case, hypothetical scenarios** and sensitivity analysis

| **Model assumptions** | **Base case & hypothetical scenarios** | **Sensitivity analysis** | | | | | | | |
| --- | --- | --- | --- | --- | --- | --- | --- | --- | --- |
|  |  | *Demographic effect* | *Compression of disability* | *Informal care shift* | *Institutional care shift* | *Carer-blind* | *Cost-pressure* | *Cost-containment* | *Income elasticity* |
| Population size & structure | Base case & hypothetical scenario 1: Based on government’s *Population Projections 2004-2036*  Hypothetical scenarios 2 & 3:  Constant throughout 2004-2036 at baseline level | Based on government’s *Population Projections 2004-2036* | | | | | | | |
| Utilisation | Base case & hypothetical scenarios 1-3:  Constant age-sex-specific predicted probabilities for each service throughout 2004-2036 | Household composition:  70-74, M: 30% living alone by 2036; 70-74, F: 40% living alone by 2036  Other age groups scaled pro rata as per 2004  Changes implemented evenly over the intervening years  Marital Status:  70-74, M: 40% separated/ divorced/ widowed; 10% single; 50% married by 2036  70-74, F: 65% separated /divorced/ widowed; 5% single; 30% married by 2036  Other age-sex groups scaled pro rata as per 2004  Changes implemented evenly over the intervening years | Brookings:  For every 1 year increase in life expectancy, age-sex-specific disability rates would advance to the corresponding age-sex strata by 1 year  Double-Brookings:  For every 1 year increase in life expectancy, age-sex-specific disability rates would advance to the corresponding age-sex strata by 2 years  Half-Brookings:  For every 1 year increase in life expectancy, age-sex-specific disability rates would advance to the corresponding age-sex strata by 0.5 year | To non-institutional care:  0.5, 1, 2% pa  Matching scheme 1:  MDS 1-7 🡪 Home care  Matching scheme 2:  MDS 1, 2,  5-7 🡪 Day care;  MDS 3-4 🡪 Home care  To institutional care:  0.5, 1, 2% pa  Matching scheme 1: MDS 1-4 🡪 Home for the Aged;  MDS 5-6 🡪 Care and Attention home;  MDS 7 🡪 Nursing home  Matching schemes 2 & 3:  Same services as scheme 1 but limited to government subvented services / private services | To non-institutional care:  1, 2, 3% every 10 years  Matching scheme 1:  MDS 1-7 🡪 Home Care  Matching scheme 2:  MDS 1, 2,  5-7 🡪 Day care;  MDS 3-4 🡪 Home care  Matching schemes 3 & 4:  Same as scheme 1 but limited to government subvented services / private services  Matching schemes 5 & 6: Same as scheme 2 but limited to government subvented services / private services | All age-sex strata 100% living alone by 2036  Changes implemented evenly over the intervening years | Constant age-sex-specific predicted probabilities for each service throughout 2004-2036 | | Scenario 1:  Income elasticity = 0.25  Scenario 2:  Income elasticity = 0.5  Scenario 3:  Income elasticity = 1 |
| Unit cost | Base case 1 & hypothetical scenario 2:  Institutional services = 2.44%;  HA services = 4%;  Non-institutional services = 3.5%;  SWD allowance benefits = 2%  Hypothetical scenarios 1 & 3:  Institutional services = 0%;  HA services = 0%;  Non-institutional services = 0%;  SWD allowance benefits = 0% | Institutional services = 2.44%;  HA services = 4%;  Non-institutional services = 3.5%;  SWD allowance benefits = 2% | | | | | Scenario 1:  Institutional services = 2.94%;  HA services = 4.5%;  Non-institutional services = 4%;  SWD allowance benefits = 2.5%  Scenario 2:  Institutional services = 3.44%;  HA services = 5%;  Non-institutional services = 4.5%;  SWD allowance benefits = 3% | Scenario 1:  Institutional services = 1.94%;  HA services = 3.5%;  Non-institutional services = 3%;  SWD allowance benefits = 1.5%  Scenario 2:  Institutional services = 1.44%;  HA services = 3%;  Non-institutional services = 2.5%;  SWD allowance benefits = 1% | Institutional services = 2.44%;  HA services = 4%;  Non-institutional services = 3.5%;  SWD allowance benefits = 2% |
| *No. of Scenarios* | *4* | *2* | *3* | *15* | *18* | *1* | *2* | *2* | *3* |

M=Male; F=Female; MDS=validated Chinese version of Minimum Data Set-Home Care; SWD=Social Welfare Department
